# The relationship between body mass index and sexual function in infertile women: A cross-sectional survey

**Published:** 2014-03

**Authors:** Safieh Jamali, Hossein Zarei, Athar Rasekh Jahromi

**Affiliations:** 1 *Department of Midwifery, Faculty of Nursing and Midwifery, Jahrom University of Medical Sciences, Jahrom, Iran.*; 2 *Department of Physical Education, Islamic Azad University, Jahrom, Iran.*; 3 *Department of Obstetrics and Gynecology, Jahrom University of Medical Sciences, Jahrom, Iran.*

**Keywords:** *Infertility*, *FSFI*, *Body mass index*, *Obesity*

## Abstract

**Background:** Infertility as the bitterest life experience can affect sexual function. Many studies have shown agitation, depression, marital dissatisfaction, and sexual dysfunction as the psychological outcomes resulting from infertility. Many factors, including body mass index, influence the female sexual function.

**Objective:** This study aimed to assess the prevalence of female sexual dysfunction and the relationship between sexual function and body mass index in the Iranian infertile women who had attended the infertility clinic.

**Materials and Methods: **This cross-sectional study was conducted on 502 infertile women who had attended Honoree clinic, Jahrom in Iran between April 2012 and December 2012. The infertile cases were classified into three groups according to the body mass index: 20-24.9 (Group I), 25-29.9 (Group II), and >29.9 and above (Group III). In addition, Female sexual function index (FSFI) questionnaire was used in order to assess the sexual problems. Finally, the data were analyzed by descriptive statistics, ANOVA and Student’s *t*-test.

**Results: **The mean age of women was 30.95±6.80 years. The results showed that 430 subjects (87.1%) had sexual dysfunction. Furthermore, the rate of sexual dysfunction among the infertile women was reported as 23.30%, 31.47%, and 45.23% in groups I, II, and III, respectively. Considering body mass index, FSFI score was 21.65±1.70 in the women with normal weight, 18.08±1.52 in overweight women, and 12.21±3.62 in obese women and the difference was statistically significant (p<0.001).

**Conclusion:** The prevalence of sexual dysfunction was quite high in infertile women, which might be due to the lack of knowledge about marital issues and lack of training in the society. If body mass index is too high, it can have a great effect on fertility. In this study, being overweight and obese based on body mass index had a negative effect on the infertile woman’s sexual function.

## Introduction

Infertility is defined as the inability to conceive after 12 months of unprotected sexual intercourse (1). World Health Organization has estimated that there are 50-80 million infertile couples worldwide (2). Up to now, only a few studies have been performed on estimation of infertility among the Iranian couples. The prevalence of primary infertility among the Iranian women aging 19-49 years was 24.9% according to a study conducted in 2004 (3)‎. In contrast, a more recent population-based study revealed that 8% of the Iranian couples suffered from infertility (4). Nowadays, infertility is considered as a social concern which can lead to the couples’ psychological imbalance, divorce, and relationship break (5, 6). Thus, in an international conference in Bangkok in 1988, infertility was described as a global health problem with physical, psychological, and social dimensions (7). Besides, in the international conference on population and development held in Cairo in 1994, it was proposed as a factor which seriously damages the reproductive health and all the countries were appointed to consider infertility in their reproductive health programs (8, 9).

Fertility status is one of the most important variables affecting marital as well as sexual satisfaction. A large number of studies have shown that infertility results in psychological disorders, including marital dissatisfaction and sexual dysfunction (10). Sexual satisfaction is mainly affected by the consequences of infertility, such as decrease of self-esteem, depression, worry, and sexual relationship with failure in reproduction (7). Overall, sexual issues and disorders are among the basic points in evaluation of an infertile couple and a desirable sexual relationship can increase the probability of reproduction (11, 12). The link between infertility and sexuality is complex and bidirectional: infertility can be considered either as a cause or a consequence of sexual dysfunction (13). 

Sexual function disorders that are more common in women include sexual desire disorders, sexual arousal disorders, orgasmic disorders, and sexual pain disorders (14, 15). The prevalence of female sexual dysfunction (FSD) has been reported as 43% in the United States of America (16). Although there are published reports regarding FSD, the accurate incidence of FSD among the infertile women is unknown (17). The causal factors of infertility, including desire, arousal, orgasm, and pain disorders, can lead to limited or avoided sexual activity, especially around the ovulation time. Studies have shown infertility has a significantly greater effect on female’s sense of sexual identity than do other stressors (18). 

Women may also experience emotional states that are known causative factors of sexual dysfunction, such as depression, anxiety, and lowered self-esteem (19). During the recent years, researchers have tried to apply scientific methods for the necessary conformities as well as compatibilities and elimination of medical problems; however, the infertile individuals’ sexual problems have been less taken into account. Overall, productivity is of great social as well as cultural value in Iran; nevertheless, insufficient attention is paid to the sexual issues in this country and more information should be provided regarding this important health priority. 

In general, a large number of factors can be effective in marital satisfaction and various researchers have expressed different factors according to their specific attitude and their research findings (20). Some studies have shown the relationship among obesity, depression, low self-esteem, and sexual problems (21, 22). However, a limited number of studies have been conducted on the relationship between sexual function and Body Mass Index (BMI) of infertile women in Iran. Therefore, the present study aims to evaluate the relationship between sexual function and BMI among the infertile women.

## Materials and methods

This cross-sectional survey was conducted on infertile women referring to Honoree clinic, in Jahrom, south of Iran between April 2012 and December 2012. The study was approved by the Ethics Committee of Jahrom University of Medical Sciences. The patients were classified as infertile according to the accepted medical definition of infertility (i.e., failure to conceive after 12 months of unprotected sexual intercourse) by the gynecologists. The study samples were selected among the infertile women referring to the infertility clinic through convenience sampling. Two midwifery specialists trained the study subjects about how to complete the questionnaires and after signing written informed consents, the participants filled out the questionnaires. Overall, 604 patients were recruited into the study; however, 102 ones did not complete the questionnaires. Therefore, the study was conducted on 502 subjects. 

This questionnaire consisted of two parts. The first part included the demographic variables; i.e., age, husband’s age, age difference between the couples, education level, occupation, height, and weight. On the other hand, the second part aimed to assess sexual function by female sexual function index (FSFI). Using a portable stadiometer, the researcher measured the women’s height and weight without shoes and with light indoor clothing. Then, the subjects' BMI was calculated as weight/height^2^ (kg/m^2^). The inclusion criteria of the study were being infertile women having no children or stepchildren after one year of regular, unprotected intercourse. Besides, all the participants were sexually active, defined as having engaged in sexual intercourse with a partner in the past 4 weeks.

On the other hand, the exclusion criteria of the study were having family disputes during the recent week, having physical problems of spinal cord injury, mutilation, paralysis, and limb deformity, having psychological problems, having medical diseases, such as cardiovascular and pulmonary disorders, hyperthyroidism, hypothyroidism, epilepsy, and diabetes, having experienced stressful events, such as death or acute disease of close relatives and major changes in life, during the past three months, being drug or alcohol abusers, and using drugs for increasing the sexual desire.

FSFI questionnaire was developed by Rosen *et al. * This questionnaire consists of 19 questions investigating the subjects in 6 domains of sexual desire, sexual arousal, lubrication, orgasm, sexual satisfaction, and pain during intercourse (23). In this questionnaire, the questions are scored based on 0.1-5 scoring system and the score of each domain is calculated through summing up the scores of that domain’s questions and multiplying the obtained number by the multiplier factor of that domain. It should be mentioned that sexual desire is covered by questions 1 and 2, excitement by the sum of questions 2, 4, 5, and 6, lubrication by adding questions 7, 8, 9, and 10, orgasm by the sum of questions 11, 12, and 13, sexual satisfaction by adding questions 14, 15, and 16, and pain by summing up questions 17, 18, and 19. In addition, multiplier factors of 0.6, 0.4, and 0.3 are used for domains including 2, 3, and 4 questions, respectively. In general, each domain has a minimum (0-1.2/1.8) and a maximum (6). 

In addition, the sexual function total score is obtained from the sum of the scores of all the domains and is ranged from 2-36. The cut-off score used to demarcate sexual dysfunction on the total FSFI score was obtained from a validation study that compared the FSFI scores of the women with documented sexual dysfunction with those of dysfunction-free volunteers and determined a total score below 26.55 to denote sexual dysfunction (20). The cut-off scores to determine the presence of difficulties on the six domains of the FSFI were obtained from published sources (23-25). Accordingly, the scores less than 4.28 in the desire domain, less than 5.08 in the arousal domain, less than 5.45 in the lubrication domain, less than 5.05 in the orgasm domain, less than 5.04 in the satisfaction domain, and less than 5.51 in the pain domain were used to classify the participants as having difficulties in that domain.

In general, researchers translate this questionnaire to the languages of their study populations (Persian) and determine its reliability and validity, as well. Overall, FSFI questionnaire is a general standard one whose reliability and validity were determined by Rosen *et al* in a study conducted in 2000 (26). Mohammadi also performed a study in Shahed University, Iran in 2004 and confirmed the reliability as well as the validity of the questionnaire. The overall validity of the questionnaire was measured through split-half and test-retest methods and reported as 78% and 75%, respectively. The validity of the subtests was also evaluated through split-half and test-retest methods and reported as 63-75% and 70-81%, respectively (27). 

**Statistical analysis**

All the study data were statistically analyzed using the SPSS software (Statistical Package for the Social Sciences, version 11.5, SPSS Inc, and Chicago, Ill, USA). Descriptive statistics, frequency, percent, mean, standard deviation, maximum, and minimum were used in this study. Student’s T-test was used to compare the FSFI domains between primary and secondary infertility. In addition, one-way ANOVA and post-hoc tests were used to determine the relationship between FSFI and BMI scores. Besides, P-value<0.05 was considered as statistically significant.

## Results

The present study was conducted on 502 infertile women in the age range of 18-44 years old with the mean age of 30.95±6.80 years. Most of the study participants were homemakers (363, 72.3%), lived in rural areas (68.9%), and had high school degrees (188, 37.5%) (Table I). Among the infertile women, 45.2% and 54.8% had primary and secondary infertility, respectively. Comparison of the sexual function domains showed the lowest mean scores to be related to desire (0.81±0.32), arousal (2.63±0.85), orgasm (2.06±0.88), pain (3.09±1.27), satisfaction (3.44+1.09), and lubrication (4.25±1.67). 

Moreover, about 70.8% of the infertile women (355 out of 502) reported “never or almost never” having a feeling of sexual desire. Also, 64.6% of the women (324) reported “never or almost never” experiencing arousal during sexual activity. Furthermore, 60.4% of the infertile women (303 out of 502) had not experienced orgasm at all. In addition, 36.6% had no lubrication during sexual activity and 48.9% had dyspareunia during sexual activity. Using the cut-off scores, 87.1% of the study women had total FSFI scores suggestive of FSD. Moreover, domain scores suggestive of difficulties related to desire, arousal, lubrication, orgasm, poor satisfaction, and pain were prevalent in 478 (95.2%), 463, 367 (73.1%), 475 (94.6%), 382 (76.1%), and 416 (82.9%) subjects, respectively. 

The prevalence of the sexual problems has been shown in table II. According to the results, the mean and standard deviation of the FSFI score for primary and secondary infertile women was 16.18±4.22 and 16.49±5.15, respectively; however, the difference was not statistically significant. Moreover, the prevalence of sexual dysfunction was 94.9% and 100% in primary and secondary infertile women, respectively (Table III). Groups I, II, and III consisted of 122, 158, and 222 patients, respectively. The study results showed a significant difference among the three study groups regarding BMI. Considering BMI, 24.3% of the women had normal weight, 31.5% were overweight, and 44.2% were obese (Table IV). A significant relationship was observed between sexual desire as well as orgasm and the infertile women’s age (p= 0.02) Also, a significant relationship was found between sexual function and age, education level, and occupation. However, no significant relationship was observed between the females’ sexual dysfunction and duration of infertility as shown in Table I.

**Table I:** Demographic characteristics of the participants and the association between sexual function and characteristics of the infertile women (N= 502)

**Characteristics **			**Mean ± SD**		
Woman's age (year)			30.95 ± 6.80		
Husband’s age (year)			35.26 ± 7.45		
Age difference between the couples			5.59 ± 4.19		
**Categories of variables **			**n (%)**	**FSFI Score Mean ± SD**	* ** p-value**
	Age (year)				
		<40	455(90.6)	16.51 ± 4.80	p= 0.02 F= 1.40
		>40	47(9.4)	14.82 ± 4.01	
	Educational level				
		Uneducated	19 (3.7)	16.97 ± 3.76	p<0.001 F= 7.79
		Primary school	147 (29.3)	17.15 ± 4.92	
		Secondary school	188 (37.5)	17.13 ± 4.60	
		Academic	148 (29.5)	15.05 ± 4.57	
	Employment status				
		Housewife	139 (27.7)	17.16 ± 4.79	p<0.001 F= 2.51
		Employed	363 (72.3)	14.23 ± 3.94	
	Infertility duration (year)				
		<5	140 (27.9)	16.02 ± 4.82	p= 0.56 F= 0.58
		5-10	287 (57.2)	16.42 ± 4.69	
		>10	75 (14.9)	16.71 ± 4.88	
	Husband's education				
		None	18 (3.6)		
		Primary school	165 (33.2)		
		High school	162 (32.5)		
		Academic	153 (30.7)		
	Husband's occupation				
		Employed	130 (25.9)		
		Non-employed	372 (74.1)		

* p-value: ANOVA and Student’s *t*-test: between female sexual function index score and characteristics of the infertile women

**Table II:** The prevalence of sexual problems in infertile women (N= 502)

**Domain**	**sexual dysfunction **	**Normal sexual function**
Desire	478 (95.2)	24 (4.8)
Arousal	463 (92.2)	39 (7.8)
Lubrication	367 (73.1)	135 (26.9)
Orgasm	475 (94.6)	27 (5.4)
Satisfaction	382 (76.1)	120 (23.9)
Pain	416 (82.9)	86 (17.1)
Total Score	430 (87.1)	72 (12.9)

**Table III:** FSFI questionnaire scores in primary and secondary infertile women

**Sexual function domains **	**Primary infertility** **(n=275)**	**Secondary infertility** **(n=227)**	* ** p-value**
Libido	0.80 ± 0.30	0.82 ± 0.34	0.62
Sexual arousal	2.64 ± 0.69	2.71 ± .97	0.37
Orgasm	2.07 ± 0.83	2.05 ± 0.92	0.75
Lubrication	4.23 ± 1.74	4.27 ± 1.62	0.75
Sexual satisfaction	3.35 ± 0.99	3.51 ± 1.16	0.10
Pain	3.06 ± 1.29	3.11 ± 1.25	0.64
Total number of sexual function	16.18 ± 4.22	16.49 ± 5.15	0.45
Sexual dysfunction N (%)	261 (94.9%)	227 (100%)	

Numbers are presented as mean±SD.

* p-value: Student’s *t*-test: between primary and secondary infertility group

**Table IV:** FSFI scores according to body mass index in infertile women

**Domain**	**BMI 20-24.9 (Normal)** **(n=122)**	**BMI 25-29.9 (Overweight)** **(n=158)**	**BMI >29.9 (Obese)** **(n=222)**	**p-value** *	**Total** **(** **n=502)**
Desire	1.05 ± 0.27	0.86 ± 0.27	0.64 ± 0.29	p<0.001	0.81 ± 0.32
Arousal	3.23 ± 0.76	2.82 ± 0.61	2.27 ± 0.85	p<0.001	2.68 ± 0.85
Lubrication	5.96 ± 0.71	4.87 ± 1.06	2.87 ± 1.22	p<0.001	4.25 ± 1.67
Orgasm	2.90 ± 0.60	2.31 ± 0.64	1.42 ± 0.66	p<0.001	2.06 ± 0.88
Satisfaction	4.37 ± 0.57	3.85 ± 0.61	2.63 ± 1.00	p<0.001	3.44 ± 1.09
Pain	4.12 ± 0.98	3.34 ± 1.08	2.35 ± 1.05	p<0.001	3.09 ± 1.27
Total Score	21.65 ± 1.70	18.08 ± 1.52	12.21 ± 3.62	p<0.001	16.35 ± 4.75

Numbers are presented as mean±SD.

* p-value ANOVA test: between first, second, and third group

## Discussion

Sexual activity is one of the most important parts of women’s life (28). In addition, various factors, including physiological factors, psychological factors, medications, infertility, lifestyle, and relationships, are effective in occurrence and progress of sexual disorders in women (29). Obesity, urinary incontinence, employment status, gynecological operations, and education level seem to have a role in sexual dysfunction, as well (30-32). The results of the present study showed the prevalence of FSD to be 87.1% among the infertile Iranian women. This study also revealed that the women's BMI was significantly correlated with their sexual function.

Rosen *et al* conducted a study on normal women and reported their mean of sexual function as 30.5% (26). Up to now, various studies have been performed on the females’ sexual function both inside and out of Iran. For instance, Zeighami *et al* found 64% lack of sexual desire and 48% decreased motivation in the women suffering from cancer (33). Moreover, Yekeh Fallah conducted a study in Ghazvin and reported the prevalence of sexual disorders as 89% among the married women, with sexual desire disorder being the most prevalent one (41.1%) (34). 

In our study, the most common sexual problems in infertile females were decreased libido (95.2%) and anorgasmia (94.6%). Holbert also estimated the prevalence of sexual disorders as 40% and mentioned sexual desire disorders as the most prevalent sexual problem among the women (35). Audu found sexual dysfunction as a prevalent problem among the infertile women (36). In addition, Monga *et al *showed that infertile women had less sexual satisfaction and more sexual problems in comparison to the fertile ones(37). In the study conducted by Tayebi *et al* in Yazd, sexual desire disorder (80.7%) and anorgasmia (83.7%) were shown as the most prevalent sexual disorders among the infertile women(38) . 

Also, in the study performed by Jain on the infertile women, 55%, 28%, and 14% of the subjects showed dyspareunia, reduction of sexual desire, and orgasm disorders, respectively, which is in line with the results of the present study (39). Jindal *et al* evaluated 200 Indian infertile women and showed that decreased frequency of intercourse and anorgasmia were the most common problems (40). Furthermore, Millheiser *et al* investigated FSFI scores in 119 infertile and 99 healthy women aging between 18 and 45 years. They detected sexual dysfunction in 25% of the fertile and 40% of the infertile subjects. They also found that the scores of desire and arousal parameters were significantly lower in the infertile group compared to the fertile group (41).

Sexual dysfunction was reported by Audu *et al* as one of the common problems in females. Besides, they reported the prevalence of frigidity, dyspareunia, arousal problems, and orgasm difficulty as 78.4%, 57.7%, 20.6%, and 20.6%, respectively (36). Hurwitz *et al* conducted a study on 40 couples with primary infertility. That study revealed increased sexual dysfunction in 50% of the females during the fertile phase of the menstrual cycle and loss of libido as the leading cause of dysfunction (42). Overall, the results of various studies have shown sexual desire disorders as the most prevalent sexual disorder among the women. Moreover, Shir Mohammadi stated that females’ sexual desire was not affected by organic factors; rather, it was influenced by self-confidence, previous sexual experiences, strong emotional relationships, hormones, and psychological diseases (43) 

It seems that sexual desire disorder in infertile women is caused by lack of free sexual activity, purposefulness of the sexual relationship, and the need for planned intercourse. Our findings also suggest that infertility impacts the women’s sexual function. The findings of the current study revealed a statistically significant relationship between age and sexual desire as well as orgasm disorders. Age is one of the factors affecting FSD. Ponholzer *et al* investigated the risk factors and prevalence of sexual dysfunction in 703 Australian women and showed that 22% and 39% had sexual desire and orgasm disorders, respectively. Also, as the age increased, these problems increased, as well (44). The present study also showed that as the age increased, sexual and orgasm disorders increased. 

In the same line, Fani Saberi *et al* revealed a significant relationship between orgasm and age in the women referring to Sari health and treatment centers (45). In that study, 96.4% of the women over 35 years old had sexual dysfunction. As the age increases, changes occur in the individuals’ sexual desire, body shape, and health status which, subsequently, affect their sexual function. Hartmann conducted a study on low sexual desire in middle-aged and old women and showed that as the age increased, the prevalence of sexual dysfunction particularly low sexual desire and sexual arousal disorders increased, as well. Stressors of life, background factors, such as the quality of the relationship and personality factors, previous sexual experiences, and physical as well as psychological health were among the other important prognostic factors of sexual dysfunction in that study (46).

In the current research, not paying attention to the problems, not following the problems, and accepting the problems were apparent in most of the cases with sexual dysfunction. Also, the results revealed no significant relationship between these disorders and duration of infertility treatment which shows that this important issue is not taken into account even after the infertile couples refer to the physician. The study findings showed a significant relationship between the women’s level of education and sexual dysfunction, which is consistent with other studies conducted on this issue (47-50). According to the results, the rate of sexual dysfunction increased with the increase in the level of education and the highest rate of dysfunction was observed among those with academic educational levels.

In this study, a significant relationship was found between sexual dysfunction and the women’s employment status and the highest rate of sexual dysfunction was observed among the employed women. Some studies have shown occupational stress, physical activity, and physical as well as mental exhaustion as the risk factors of sexual dysfunction and reduction of enjoyment in sexual relationships (49, 51). Comparison of the women with primary and secondary infertility revealed no significant difference between the two groups regarding the FSFI scores. The findings of the present study showed that the women with secondary infertility reported a higher prevalence of FSD compared to those with primary infertility.

The findings of a study conducted in Turkey also showed a higher prevalence of sexual dysfunction in secondary infertile women compared to those with primary infertility, which is quite considerable compared to the results of the present study (52). Furthermore, this study indicated that the obese women, with high BMI, were at a greater risk of sexual dysfunction. High BMI plays a key role as a predictor of poor sexual function. Excess weight increases the risk of reproductive problems (53). The results of the present study showed as the BMI increased, sexual dysfunction increased, as well. The findings of other investigations have also shown that the women with high BMI were more likely to have fertility problems (54-60). Previous researches indicated a relationship between infertility and lower sperm count, obesity, and lack of sexual satisfaction (61). Besides, Esposito *et al *believed that obesity was associated with both sexual and erectile dysfunction (62).

In this study, approximately 44.1% of the infertile women had BMI >29.9 and the lowest sexual function scores. In addition, higher BMI was associated with lower sexual function in infertile women. Many studies have also shown the relationship between obesity and infertility (63-65). The sex hormones imbalance may affect reproduction in women and high BMI can affect the women's hormone levels (66). Kaneshiro *et al* believed that BMI and sexuality outcomes were mixed (67). Furthermore, some studies have shown that the women who were dissatisfied with their physical appearance reported less confidence in sexual interactions (68-70). Previous studies have shown the relationship between obesity and sexual dysfunction in women, as well (71-73).

**Strong points and limitations**

One of the strong points of this study was using FSFI questionnaire which contains all the key dimensions of sexual function, has a high reliability as well as validity, and has been less used in Iranian studies. However, the noise in the environment while completing the questionnaires could affect the samples’ responses. 

Of course, a special place was considered in the infertility clinic in order to maintain the subjects’ privacy. In addition, the individuals referring to infertility clinic had a specific socio-cultural status and a considerable amount of time had to be spent for explaining the questions and obtaining accurate answers. Nevertheless, since it was the first time that these issues were discussed with them, their answers were quite honest and reliable.

## Conclusion

In this study, sexual dysfunction was quite high among the infertile women, which might be due to the lack of knowledge about marital issues, lack of training in the society, and wrong cultural beliefs. Infertility is a distinctive experience and, as a result, understanding the individuals’ thoughts and attitudes can play a critical role in understanding the psycho-social considerations of infertility. In addition, 102 patients did not complete or return the forms. This can be explained by the general Islamic community and its taboos about putting sexuality into words and confessing sexual satisfaction, especially in women.

Qualitative studies can also be beneficial in deep understanding of the socio-cultural aspects of infertility. The researchers hope that the findings of the present study can be used for developing various consultation and supportive programs for such couples. It is also necessary that the infertility specialists pay more attention to the psycho-sexual dimensions of infertility and training programs regarding marital as well as sexual skills be held by supportive groups in infertility clinics. In this way, by presenting appropriate solutions for elimination of pressure and tension in infertile individuals, major steps can be taken toward improving their quality of life. Health promotion should also focus on improving the impaired sexual function of overweight and obese women. They should be aware of the relationship between BMI and sexual dysfunction, as well.

Further studies are required to compare sexual function in fertile and infertile women, investigate the relationship between BMI and sexual function in fertile and infertile women, and determine the effects of BMI on infertility in Iranian population. As mentioned above, overweight and obesity can enhance the chance of sexual dysfunction and consequently infertility.
